# *Aspergillus* sensitisation detection using point-of-care lateral flow assay in moderate to severe asthma

**DOI:** 10.1093/mmy/myad076

**Published:** 2023-07-25

**Authors:** Ran Wang, Chris Eades, Maisie Palmer, Gareth Platt, Stephen J Fowler, Chris Kosmidis

**Affiliations:** Division of Immunology, Immunity to infection & Respiratory Medicine, School of Biological Sciences, The University of Manchester, Manchester M23 9LT, UK; Manchester University NHS Foundation Trust, Manchester Academic Health Science Centre, Manchester M23 9LT, UK; Division of Immunology, Immunity to infection & Respiratory Medicine, School of Biological Sciences, The University of Manchester, Manchester M23 9LT, UK; Manchester University NHS Foundation Trust, Manchester Academic Health Science Centre, Manchester M23 9LT, UK; Mycology Reference Centre Manchester, Manchester M23 9LT, UK; Division of Immunology, Immunity to infection & Respiratory Medicine, School of Biological Sciences, The University of Manchester, Manchester M23 9LT, UK; Division of Immunology, Immunity to infection & Respiratory Medicine, School of Biological Sciences, The University of Manchester, Manchester M23 9LT, UK; Manchester University NHS Foundation Trust, Manchester Academic Health Science Centre, Manchester M23 9LT, UK; Manchester University NHS Foundation Trust, Manchester Academic Health Science Centre, Manchester M23 9LT, UK; Division of Evolution, Infection and Genomics, School of Biological Sciences, The University of Manchester, Manchester M23 9LT, UK

**Keywords:** *Aspergillus*, ABPA, severe asthma, diagnosis

## Abstract

Allergic fungal airway diseases are associated with asthma exacerbations and poor control. However, the early identification of allergic *Aspergillus* airway diseases can be challenging, especially in resource-poor countries. We aimed to evaluate the clinical utility of the point-of-care *Aspergillus* IgG–IgM lateral flow assay in diagnosing *Aspergillus* airway diseases in patients with moderate–severe asthma. Patients with moderate–severe asthma, severe asthma with fungal sensitisation (SAFS) and allergic bronchopulmonary aspergillosis (ABPA) were recruited. Clinical information was extracted from clinical records. Blood samples were collected for serological tests. Serum samples were evaluated using *Aspergillus* immunochromatographic test (ICT). A total of 65 patients were recruited into the study, of whom 23.1% had clinical diagnosis of ABPA, 18.5% had SAFS and 58.5% had moderate-to-severe asthma who did not fit ABPA or SAFS criteria. The ICT test gave a sensitivity of 69 [95% confidence interval: 51–88]% and a specificity of 77 [60–88]% in predicting a positive *Aspergillus* IgG test. The sensitivity and specificity for a positive *Aspergillus* IgE were 77 [59–88]% and 86 [71–94]%, respectively. The majority (sensitivity: 87 [62–96]%) of patients with ABPA had positive ICT results, with a specificity of 70%. The negative predictive value was high (95 [82–99]%) with a low negative likelihood ratio (< 0.2), making it potentially useful in ruling out ABPA. The ICT assay may be valuable in ruling out ABPA in resource-limited countries where serological investigations are less feasible. The ICT assay may be particularly useful in ruling out ABPA and warrants further validation.

## Introduction

Allergic fungal airway diseases, such as severe asthma with fungal sensitisation (SAFS) and allergic bronchopulmonary aspergillosis (ABPA), are associated with asthma exacerbations and poor control.^1, 2^ ABPA is associated with lung damage including the development of bronchiectasis, pulmonary fibrosis, and progressive loss of lung function. The early identification of allergic *Aspergillus* airway diseases can be challenging.^1, 2^ Furthermore, it manifests as a clinical spectrum rather than several distinct disease entities.^3^ Diagnosing allergic fungal airway diseases requires accessibility to serological tests and imaging ^4–6^ which pose a significant challenge in resource-poor counties where fungal sensitisation in asthma is prevalent.^7^ A novel point of care *Aspergillus* IgG–IgM lateral flow assay (LDBio diagnostics, Lyons, France) ^8^ has been developed. Whilst the immunochromatographic test (ICT) requires minimal time and resources, and has demonstrated clinical usefulness in chronic pulmonary aspergillosis,^9^ its diagnostic efficiency in allergic *Aspergillus* airway diseases has been inconsistent in different settings.^10–12^ Notably, these studies either included patients without asthma,^12^ milder forms of asthma, ^11^ or a population with undefined asthma severity as the control groups,^10^ and the role of ICT assay in a more clinically relevant population remains unclear.

Therefore, we investigated the association between ICT assay response and relevant serological parameters in patients with SAFS, ABPA, and moderate to severe asthma without fungal allergy, and evaluated its diagnostic performance in a clinically relevant setting.

## Methods

Patients with moderate to severe asthma (British Thoracic Society treatment step 3 or above) attending the asthma or fungal lung disease clinics of the North West Lung Centre, Manchester University NHS Foundation Trust were eligible. All participants consented into the Manchester Allergy, Respiratory and Thoracic Surgery (ManARTS, Manchester, UK) research tissue bank (15/NW/0409). Blood samples were collected and sera either processed immediately or stored at −80°C until analysis. Clinical information was extracted from clinical records.

We defined each diagnostic group based on (1) serological evidence only and (2) clinical diagnosis. Patients were serologically defined as having ABPA if they had ever demonstrated evidence of *Aspergillus*-specific (Asp) IgE (Immunoglobulin E)> 0.34 kU_A_/ml *and* total IgE > 1000 kU_A_/ml *and* (either Asp IgG (Immunoglobulin G) > 40 mgA/l *or* blood eosinophilia > 0.5 × 10^9^ cells/l); SAFS was serologically defined as patients with severe asthma who had ever demonstrated evidence of Asp IgE > 0.34 kU_A_/ml *and* total IgE < 1000 kU_A_/ml. Clinical diagnoses of SAFS and ABPA were made by experienced specialist clinicians at the National Aspergillosis Centre and tertiary severe asthma centre (Manchester, UK) and confirmed by the authors by reviewing historical clinical notes, radiology, serial serological results, and treatment responses. Patients who did not fit ABPA or SAFS criteria were categorised as controls.

Total IgE, Asp IgG, Asp IgE, and blood eosinophil counts were measured either as part of routine clinical care or collected through the ManARTS tissue bank. ImmunoCAP EIA (ThermoFisher Scientific, Waltham, MA, USA) was used for Asp IgG and IgE (Asp IgE cutoff > 40 mgA/l and Asp IgE cutoff > 0.34 kUA/ml). LDBio ICT assay was interpreted both visually and digitally using a lateral flow reader (Qiagen ESEQuant LR3 [Lake Constance, Germany]), a fluorescence reader that expresses results as a peak height in mV.

Correlations between serological tests and ICT were evaluated using Spearman’s Rank tests. Comparisons between categorical variables were performed using *χ*^2^ test, or Fisher’s exact test, as appropriate. Diagnostic performance was assessed using descriptive statistics and the area under the receiver operating characteristic curve (AUROCC). All analysis was performed in R (version 4.1.1).

## Results

Of 65 patients, 23.1% had clinical diagnosis of ABPA, 18.5% had SAFS and 58.5% moderate-to-severe asthma patients (without ABPA or SAFS). The agreement between serological and clinical diagnosis was 95.4%. Three patients who had fulfilled the serological criteria for ABPA had alternative clinical diagnoses (*n* = 2 as SAFS and *n* = 1 control; all had absence of bronchiectasis on computer tomography and significant co-sensitisation to multiple allergens). All data presented refer to clinical diagnosis unless otherwise specified.

A total of 40% of patients with ABPA and 50% with SAFS were prescribed long-term oral corticosteroids (OCS), and 47/40%, respectively anti-fungal therapies at the time of the ICT (Table 1).

**Table 1.** Baseline characteristics.

Diagnosis (*n*)	ABPA (15)	SAFS (12)	Moderate–severe asthma (38)
Age, mean (SD) yrs	61.5 (8.6)	57.1 (14.8)	52.6 (14.8)
Gender, *n* (%) male	8 (53.3%)	5 (41.7%)	9 (23.7%)
OCS maintenance, *n* (%)	6 (40%)	5/10 (50%)	14 (36.8%)
Anti-fungal treatment, *n* (%)	7 (46.7%)	4 (40%)	6 (15.8%)*
*Serology at the time of ICT test*			
Asp IgG, median (IQR) mgA/l	53.0 (38.5–78)	51.5 (15.8–66.8)	27.5 (14.0–55.0)
Asp IgE, median (IQR) kU_A_/ml	55.5 (25.0–64.8)	1.8 (0.7–2.7)	0 (0–0.1)
Blood eosinophils median (IQR) x10^9^ cells/l	0.34 (0.22–0.54)	0.19 (0.12–0.38)	0.2 (0.08–0.37)
Total IgE, median (IQR) IU/ml	2413 (1701–4513)	210 (120–305)	76.2 (33.7–257)
Positive LDBio (visual interpretation), *n* (%)	13 (86.7%)	7 (58.3%)	8 (21.1%)^¥^

* Antifungal started for reasons other than allergic *Aspergillus* airway diseases; *n* = 2 who had antifungal therapy initiated without clear evidence of fungal involvement in the clinical condition. *n* = 3 had co-existing *Aspergillus* bronchitis and *n* = 1 had antifungal treatment for *Candida* sensitisation.

ABPA, allergic bronchopulmonary aspergillosis; IQR, interquartile range; SAFS, severe asthma with fungal sensitisation; and OCS, oral corticosteroids. .

¥ one patient with *Aspergillus terreus* bronchitis, with negative ICT results.

### Correlation between ICT and serological tests

**Figure 1.**
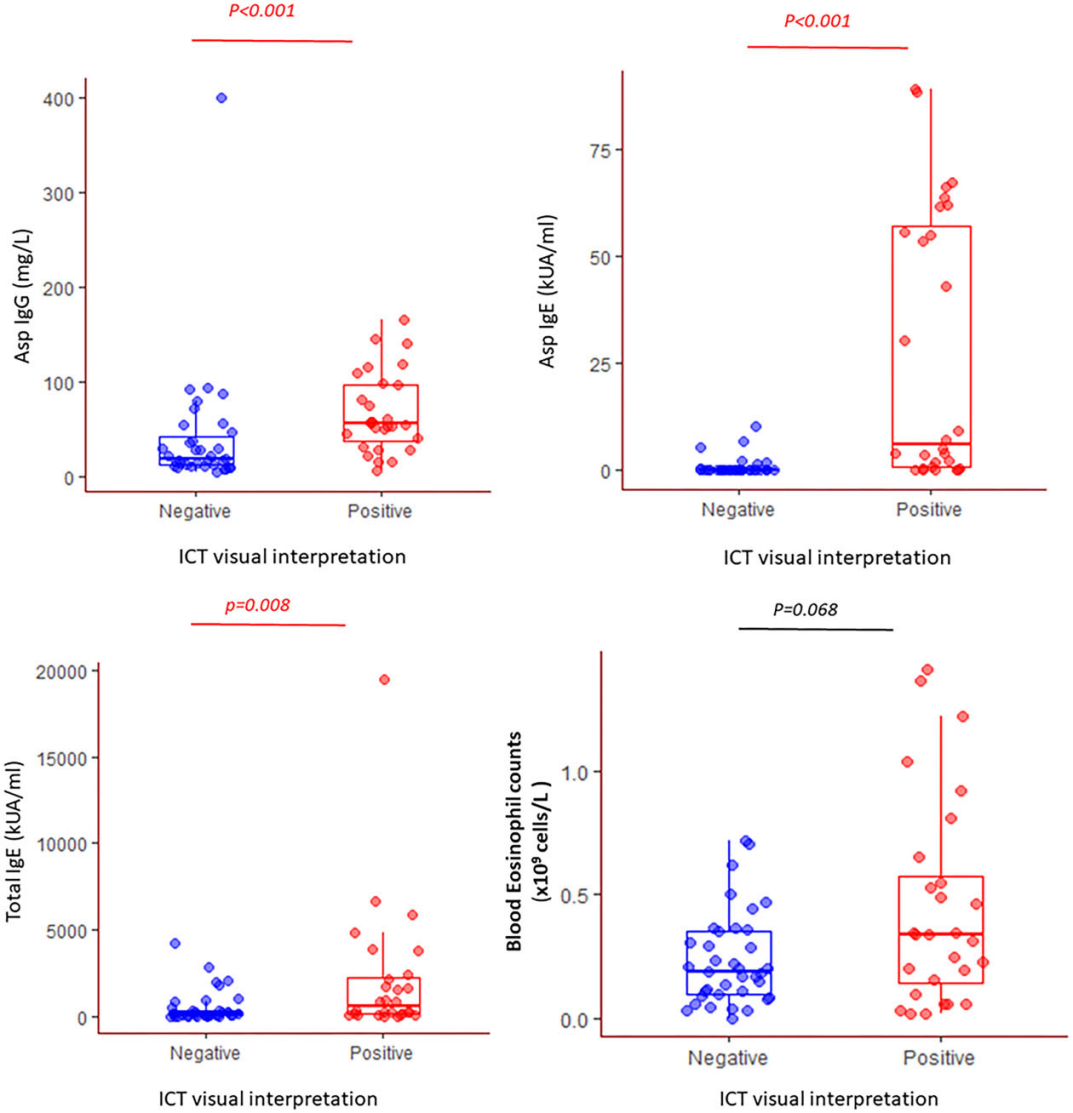
Serological test results (individual data and box-and-whisker plots) in patients with positive and negative immunochromatographic test (ICT) based on visual interpretation.

**Figure 2.**
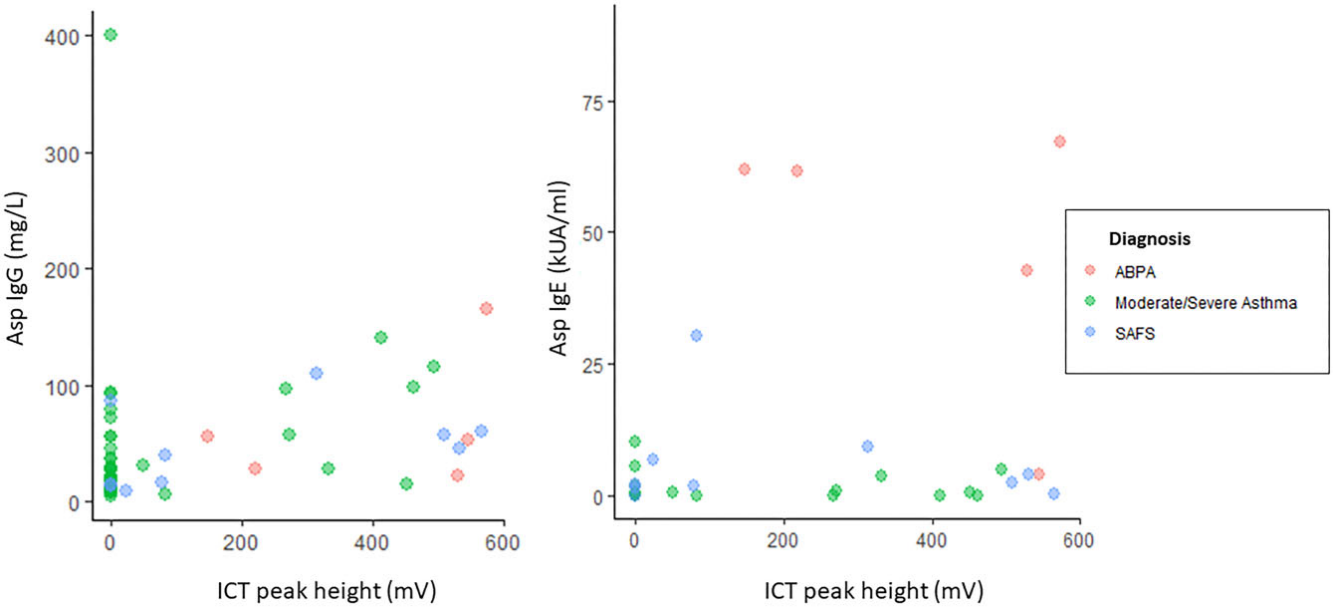
Scatter plots of ICT peak height (digital reading) versus Asp IgG (left) and Asp IgE (right).

**Figure 3.**
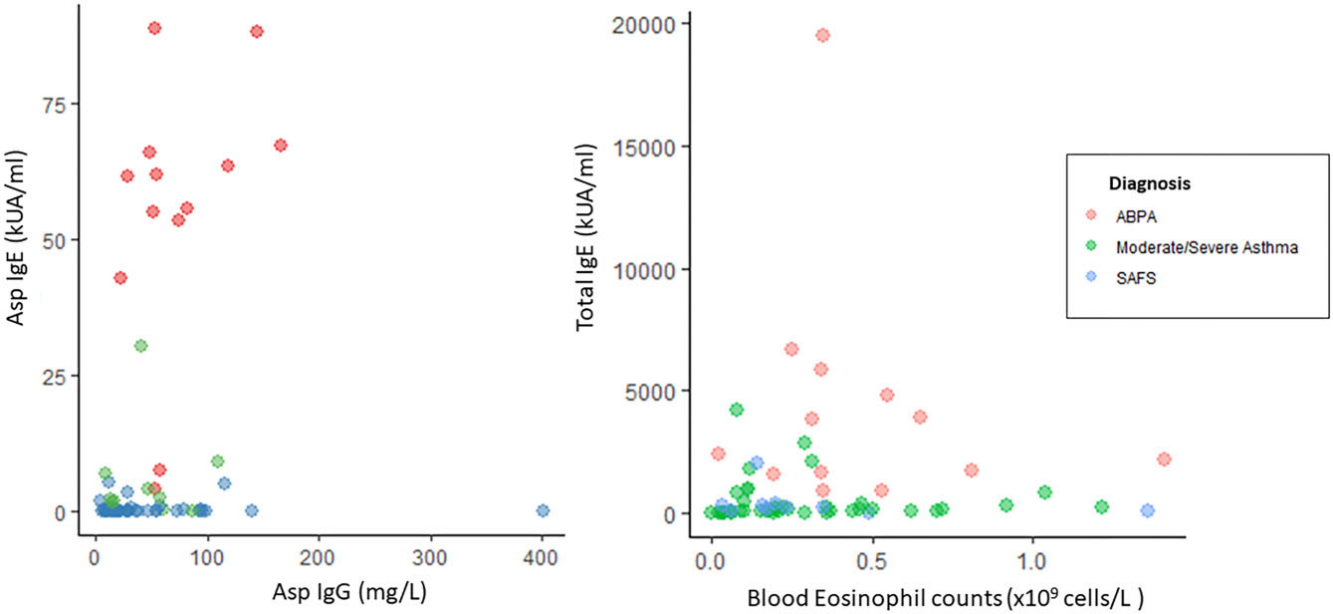
Scatter plots of Asp IgG versus Asp IgE (left) and blood eosinophils versus total IgE (right).

The median (IQR) duration between ICT and IgE tests was 49 (0–90) days, and 0 (0–52) days for Asp IgG. Asp IgG, Asp IgE, and total IgE (but not blood eosinophils) were significantly higher in patients who had positive ICT than those with negative test results based on visual interpretation (Fig. 1). A total of 56 (86.2%) patients had ICT interpreted both visually and digitally with excellent agreement (98.2%). Only one participant (serological and clinical diagnosis of ABPA with serum Asp IgG titre of 9 mgA/l) with a low ICT peak height of 23.3 mV was visually interpreted as a negative result. The peak height, determined with the lateral flow digital reader, correlated with Asp IgG (*r* = 0.40, *P *= .003) and Asp IgE (*r* = 0.58, *P *< .001) (Fig. 2) but not with eosinophil count or total IgE. Asp IgE also modestly correlated with IgG (*r* = 0.25, *P *= .05) (Fig. 3). The visual interpretation of ICT gave 73% agreement with Asp IgG and 82% agreement with Asp IgE. Blood eosinophil counts only modestly correlated with total IgE (*r* = 0.27, *P *= .029) (Fig. 3), with 69.2% agreement between the two tests.

**Figure 4.**
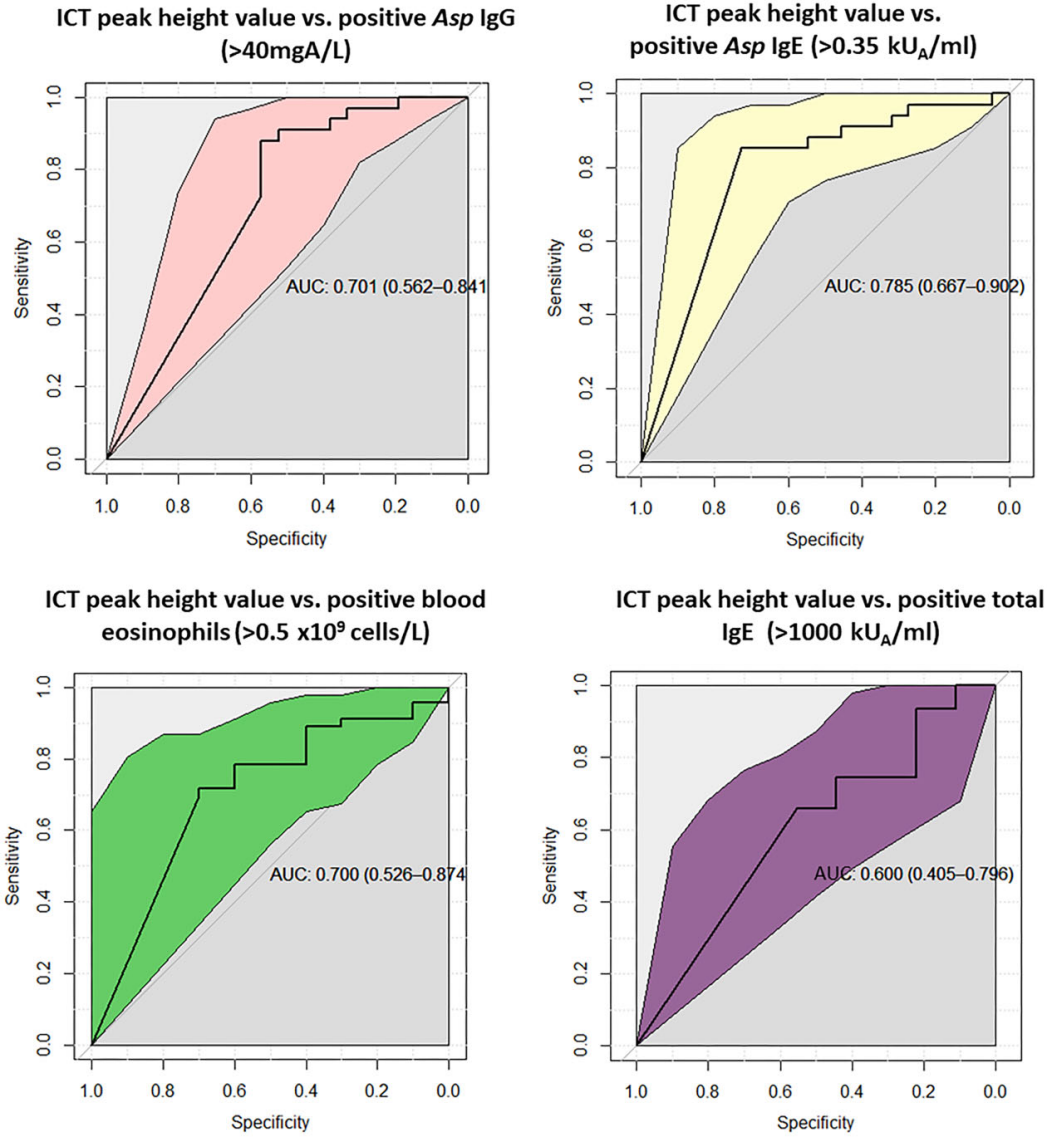
Receiver operating characteristic curves for ICT peak height to predict positivity of *Asp* IgG, *Asp* IgE, blood eosinophilia, and total IgE.

The peak height (digital interpretation) of the ICT test gave a sensitivity of 69% and specificity of 77% in predicting a positive Asp IgG test, whereas the sensitivity and specificity for a positive Asp IgE were better (77% and 86%, respectively) (Table 2). The ICT peak height values gave a mean (95% confidence interval) AUROCC of 0.70 (0.56–0.84) for a positive Asp IgG, 0.79 (0.67–0.90) for a positive Asp IgE test, 0.70 (0.53–0.87) for blood eosinophilia (> 0.5 × 10^9^ cells/l, Fig. 4), and (0.60 [0.41–0.80]) for a positive total IgE (> 1000 kU_A_/ml).

**Table 2.** Diagnostic performance of visual interpretation of LDBio assay.

	Sensitivity *N* (% [95%CI])	Specificity *N* (% [95%CI])	PPV *N* (% [95%CI])	NPV *N* (% [95%CI])	+LR	–LR
Prediction of serological tests						
High Asp IgG (> 40 mgA/l)	20/29 (69.0 [50.8, 82.7]%)	26/34 (76.5 [60.0, 87.6]%)	20/28 (71.4 [52.9, 84.7]%)	26/35 (74.3 [57.9, 85.8]%)	2.94	0.41
High Asp IgE (> 0.34 kU_A_/ml)	23/30 (76.7 [59.1, 88.2]%)	30/35 (85.7 [70.6, 93.7]%)	30/37 (81.1 [65.8, 90.5]%)	23/28 (82.1 [64.4, 92.1]%)	5.36	0.27

+LR = sensitivity/(1-specificity) and –LR= (1-sensitivity)/specificity. CI, confidence interval; LR, likelihood ratio; NPV, negative predictive value; and PPV, positive predictive value.

### The diagnostic performance of ICT

The majority (87%) of patients with a clinical diagnosis of ABPA, 58% of SAFS and 21% of patients without ABPA and SAFS had positive ICT results. As the false negative rate was low for ABPA and the negative predictive value was high (95%) with a low negative likelihood ratio (< 0.2), the ICT was useful in ruling out the disease (Table 3). A negative result only modestly reduced the probability of any allergic *Aspergillus* airway disease (ABPA or SAFS) in moderate-to-severe asthma with a –LR of 0.33. Using serological definitions produced similar results (Table 3).

**Table 3.** Diagnostic performance of ICT in allergic *Aspergillus* airway diseases.

	Sensitivity *N* (% [95%CI])	Specificity *N* (% [95%CI])	PPV *N*(% [95%CI])	NPV *N*(% [95%CI])	+LR	–LR
***Clinical diagnosis***						
ABPA *versus* not ABPA	13/15 (86.7 [62.1, 96.3]%)	35/50 (70.0 [56.2, 80.9]%)	13/28 (46.4 [29.5, 64.2]%)	35/37 (94.6 [82.3, 98.5]%)	2.89	0.19
Allergic *Aspergillus* airway diseases^†^*versus* control	20/27 (74.1 [55.3, 86.8]%)	30/38 (78.9 [63.7, 88.9]%)	20/28 (71.4 [52.9, 84.7]%)	30/37 (81.1 [52.9, 84.7]%)	3.51	0.33
***Serological diagnosis***						
ABPA *versus* not ABPA	14/18 (77.8 [54.8, 91.0]%)	33/47 (70.2 [56.0, 81.3]%)	14/28 (50.0 [32.6, 67.4]%)	33/37 (89.2 [75.3, 95.7]%)	2.61	0.32
Allergic *Aspergillus* airway diseases^†^*versus* control	21/28 (75.0 [56.6, 87.3]%)	30/37 (81.1 [65.8, 90.5]%)	21/28 (75.0 [56.6, 87.3]%)	30/37 (81.1 [65.8, 90.5]%)	3.97	0.31

+LR = sensitivity/(1-specificity); −LR= (1-sensitivity)/specificity; and † = Allergic *Aspergillus* airway diseases is defined as *either* severe asthma with fungal sensitisation *or* allergic bronchopulmonary aspergillosis.

CI, confidence interval; LR, likelihood ratio; NPV, negative predictive value; and PPV, positive predictive value.

Patients who were prescribed antifungal medications more commonly had a positive ICT (70.6%) than those who were not (29.4%, *P *= .008), but no difference in the prevalence of positive ICT was found in patients who were treated with OCS compared to those without (*P *= .669).

**Figure 5.**
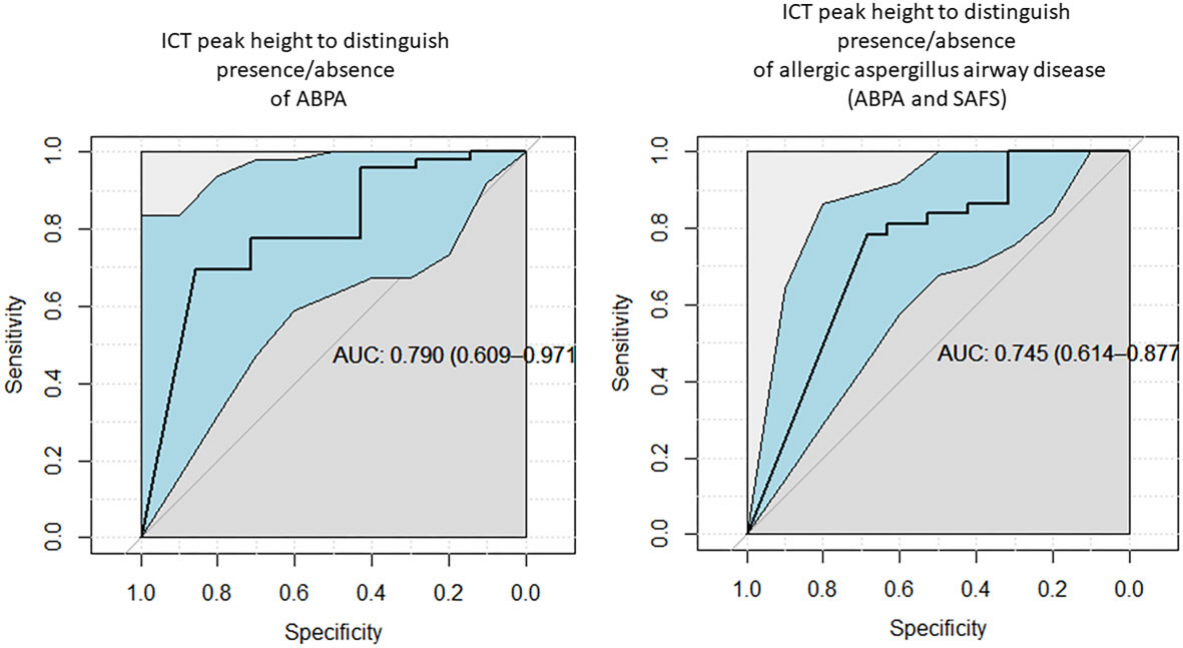
Receiver operating characteristic curves for ICT peak height to predict allergic *Aspergillus* airway disease and ABPA.

The ICT peak height values gave an AUROCC of 0.79 (0.61–0.97) for discriminating clinical diagnosis of ABPA from non-ABPA diseases and 0.75 (0.61–0.88) (Fig. 5) in discriminating ABPA and SAFS from the other diseases.

## Discussion

The ICT assay demonstrated high sensitivity and may be useful in ‘ruling out’ ABPA in high-risk asthma patients. The patient characteristics observed in this study mimic the clinical settings where patients are at risk of developing allergic *Aspergillus* airway diseases and the ICT is likely to be used. Notably, we have demonstrated the complex clinical spectrum and the overlapping serological patterns of allergic *Aspergillus* airway diseases in moderate-to-severe asthma patients. Whilst the probability of having a positive ICT result increased from SAFS to ABPA, approximately 20% of moderate to severe asthma patients who did not satisfy the SAFS or ABPA diagnostic criteria had false positive results. This is likely due to the heterogeneity of this group (Table 1). Interestingly, of these, the majority of patients (eight out of nine) had either a positive Asp IgG and/or Asp IgE test. Given that the prevalence of ABPA is around 3% among asthma patients,^13^ the positive predictive value is poor for a screening test in a general asthma population.

Consistent with the previous findings by Hunter *et al*., a modest correlation was found between Asp IgG and IgE titres.^10^ However, the correlation between ICT and Asp IgE and IgG was not previously reported.^10^ Kwizera *et al*. found poor clinical utility as a screening tool for ABPA amongst patients with varying severities of asthma, but a higher Asp IgE was found also reported in patients with a positive ICT result. It is unclear how the time between ICT and the routine clinical serological tests influenced the results. In the current study, the time between the ICT assay and serological tests was short (mostly within 3 months for Asp IgE and 2 months for Asp IgG), potentially explaining the differences observed in the studies. Whilst the ICT test detects IgG and IgM class anti-*Aspergillus* antibodies, a better correlation and prediction to the Asp IgE serological tests in patients with a high risk of allergic *Aspergillus* airway diseases was found. This association could be confounded by the correlations between Asp IgG and Asp IgE in this population. Furthermore, the ICT uses a homogeneous antigen sandwich format to detect *Aspergillus* antibody and it has been suggested that it may detect immunoglobulin classes other than IgG and IgM,^10^ and warrants further investigation. ICT gave a limited predictive ability to be clinically useful as a surrogate test for Asp IgG, but a moderately low –LR (likelihood ratio) (0.27) and high negative predictive value (∼82%) in predicting Asp IgE, suggesting that it may have a role in determining *Aspergillus* sensitisation. Digital interpretation of ICT did not show advantages over visual readings.

### Strengths and limitations

To our knowledge, our study is the first to test ICT in a clinically relevant setting, where allergic *Aspergillus* airway diseases are common and diagnostic decisions are often needed. However, we had a relatively small sample size and hence it is imperative to validate our findings in a larger cohort. We compared the ICT performance in clinical ABPA diagnosis and those based on historical serological evidence only and demonstrated similar results. The clinical diagnoses of ABPA and SAFS in the current study were confirmed retrospectively with all clinical data and historical serological tests. Whilst this approach may be more robust in the classification of diagnostic groups, future studies should aim to prospectively evaluate the diagnostic efficiency of ICT in this setting. Furthermore, we have found that even in experienced specialist centres, differentiating ABPA, SAFS, and other allergic *Aspergillus* airway diseases may be challenging, and the diagnostic label are often interchangeably used over time in clinical practice. This may be due to the longitudinal variations in disease progression/remission and the associated serological test results. The serological classifications of ABPA and SAFS using dichotomous cutoff values were also limited in describing all the phenotypical differences amongst the spectrum of allergic *Aspergillus* airway diseases; this should be investigated in future research.

## Conclusion

The ICT assay may be valuable in ruling out ABPA in resource-limited countries where serological investigations are less feasible. Patients with a positive test and a clinical suspicion of ABPA should then have further investigations to diagnose ABPA. However, our study is limited by the small sample size and should be further validated in a larger cohort before clinical implementation.

## References

[1] Wardlaw AJ , Rick E, Ozyigit LP et al. New perspectives in the diagnosis and management of allergic fungal airway disease. J Asthma Allergy. 2021; 14: 557–573. 34079294 10.2147/JAA.S251709 PMC8164695

[2] Agarwal R , Sehgal I, Dhooria S et al. Allergic bronchopulmonary aspergillosis. Indian J Med Res. 2020; 151:529–549. 32719226 10.4103/ijmr.IJMR_1187_19 PMC7602921

[3] Kosmidis C , Denning DW, The clinical spectrum of pulmonary aspergillosis. Thorax. 2015; 70: 270–277. 25354514 10.1136/thoraxjnl-2014-206291

[4] Denning DW , O’Driscoll BR, Mogaboam CM et al. The link between fungi and severe asthma: A summary of the evidence. Eur Respir J. 2006; 27: 615–626. 16507864 10.1183/09031936.06.00074705

[5] Nazik Bahçecioğlu S , Turk M, Cetin GP et al. The adequacy of current diagnostic criteria for making a diagnosis of ABPA. Tuberk Toraks. 2022; 70: 141–148. 35785878 10.5578/tt.20229804

[6] Agarwal R , Chakrabrti A, Shah A et al. Allergic bronchopulmonary aspergillosis: Review of literature and proposal of new diagnostic and classification criteria. Clin Exp Allergy. 2013; 43: 850–873. 23889240 10.1111/cea.12141

[7] Kwizera R , Musaazi J, Meya DB et al. Burden of fungal asthma in Africa: A systematic review and meta-analysis. PLoS One. 2019; 14: e0216568. 31095641 10.1371/journal.pone.0216568 PMC6521988

[8] LDBio. *Aspergillus* ICT IgG-IgM assay. Available from https://ldbiodiagnostics.com/en/our-products/rapid-tests/aspergillus-ict-igg-igm/. Accessed 28/07/2023.

[9] Guo Y , Bai Y, Yang C et al. Evaluation of *Aspergillus* IgG, IgM antibody for diagnosing in chronic pulmonary aspergillosis: A prospective study from a single center in China. Medicine (Baltimore). 2019; 98: e15021. 31008929 10.1097/MD.0000000000015021 PMC6494343

[10] Hunter ES , Page ID, Richardson MD et al. Evaluation of the LDBio *Aspergillus* ICT lateral flow assay for serodiagnosis of allergic bronchopulmonary aspergillosis. PLoS One. 2020; 15: e0238855. 32976540 10.1371/journal.pone.0238855 PMC7518618

[11] Kwizera R , Bongomin F, Olum R et al. Evaluation of an *Aspergillus* IgG/IgM lateral flow assay for serodiagnosis of fungal asthma in Uganda. PLoS One. 2021; 16: e0252553. 34048497 10.1371/journal.pone.0252553 PMC8162618

[12] Piarroux RP , Romain T, Martin A et al. Multicenter evaluation of a novel immunochromatographic test for anti-*Aspergillus* IgG detection. Front Cell Infect Microbiol. 2019; 9: 12. 30766842 10.3389/fcimb.2019.00012 PMC6365447

[13] Denning DW , Pleuvry A, Cole DC, Global burden of allergic bronchopulmonary aspergillosis with asthma and its complication chronic pulmonary aspergillosis in adults. Med Mycol. 2013; 51: 361–370. 23210682 10.3109/13693786.2012.738312

